# RNA-seq analysis of amygdala tissue reveals characteristic expression profiles in schizophrenia

**DOI:** 10.1038/tp.2017.154

**Published:** 2017-08-15

**Authors:** X Chang, Y Liu, C-G Hahn, R E Gur, P M A Sleiman, H Hakonarson

**Affiliations:** 1 Center for Applied Genomics, The Children’s Hospital of Philadelphia, Philadelphia, PA, USA; 2 Neuropsychiatric Signaling Program, Department of Psychiatry, Perelman School of Medicine, University of Pennsylvania, Philadelphia, PA, USA; 3 Neuropsychiatry Section, Department of Psychiatry, Perelman School of Medicine, University of Pennsylvania, Philadelphia, PA, USA; 4 Division of Human Genetics, Department of Pediatrics, The Perelman School of Medicine, University of Pennsylvania, Philadelphia, PA, USA

## Abstract

The amygdala brain region has been implicated in the pathophysiology of schizophrenia through emotion processing. However, transcriptome messages in the amygdala of schizophrenia patients have not been well studied. We used RNA sequencing to investigate gene-expression profiling in the amygdala tissues, and identified 569 upregulated and 192 downregulated genes from 22 schizophrenia patients and 24 non-psychiatric controls. Gene functional enrichment analysis demonstrated that the downregulated genes were enriched in pathways such as 'synaptic transmission' and 'behavior', whereas the upregulated genes were significantly over-represented in gene ontology pathways such as 'immune response' and 'blood vessel development'. Co-expression-based gene network analysis identified seven modules including four modules significantly associated with 'synaptic transmission', 'blood vessel development' or 'immune responses'. Taken together, our study provides novel insights into the molecular mechanism of schizophrenia, suggesting that precision-tailored therapeutic approaches aimed at normalizing the expression/function of specific gene networks could be a promising option in schizophrenia.

## Introduction

Schizophrenia is a brain disorder, characterized by abnormal social behavior and failure to differentiate real from unreal. Schizophrenia typically presents with symptoms of unclear or confused thinking, lack of reality recognition, auditory hallucinations, reduced social engagement and attenuated emotional expression.

The amygdala region has an important role in the processing of emotions and has been implicated in the pathophysiology of schizophrenia.^1, 2, 3, 4^ As perturbations of emotional responsiveness present a major clinical feature of schizophrenia, dysfunction within the amygdala region of the brain may contribute to the behavioral phenotypes of patients diagnosed with schizophrenia. For example, functional neuroimaging studies have demonstrated altered amygdala activity as an underlying factor of emotional dysfunction in schizophrenia.^5, 6, 7^ Reduced amygdala volumes have also been demonstrated among patients with early-course schizophrenia.^8^

To explore the molecular mechanism underlying schizophrenia, a number of transcriptome studies using microarray or RNA sequencing (RNA-seq) techniques have been conducted, using tissues from different brain regions, including the superior temporal gyrus,^9^ dorsolateral prefrontal cortex,^10, 11, 12, 13^ cerebellar cortex,^14^ hippocampus^15^ and the anterior cingulate cortex.^16^ Most of the transcriptome studies have pointed to the abnormal activation of the immune system in the brain of schizophrenia patients.^10, 11, 13, 15, 17, 18^ Gene markers such as *SERPINA3*, *IFITM1*, *IFITM2* and *IFITM3* have been consistently replicated in multiple studies.^9, 10, 11, 13, 15, 18^ However, gene-expression alterations in the amygdala of schizophrenia patients have not been thoroughly studied. Although a previous transcriptome study of amygdala using microarray chips suggested that genes from the cytomatrix-active zone may be upregulated,^19^ our understanding of the overall gene dysregulation in the amygdala of schizophrenia patients is still unclear.

In this study, we investigated gene-expression profiling in the amygdala tissues from 22 patients with schizophrenia and 24 non-psychiatric controls, using RNA-seq. We uncovered several dysregulated genes and pathways that are associated with the pathophysiology of schizophrenia. We further performed weighted gene correlation network analysis (WGCNA) to construct a gene co-expression network and identify functional modules of highly correlated genes.^20^ Our study provides convergent support for the established role of immune system in schizophrenia, as well as novel insight into the specific molecular mechanisms involving synaptic transmission in schizophrenia, with potential implications for the development of novel treatment for patients diagnosed with schizophrenia.

## Materials and methods

### Post-mortem brain samples

Amygdala of post-mortem brain was obtained from the Lieber Brain bank (http://www.libd.org) with consent. The Research Ethics Board of CHOP approved the study, and written informed consent was obtained from all subjects. Cases in this study included nine, seven, five and one patients with diagnosis of undifferentiated, disorganized, paranoid schizophrenia and schizoaffective disorder, respectively. Control subjects were individuals without psychiatric diagnoses. The post-mortem interval, age and gender of cases and controls were well matched (Supplementary Table S1). Non-parametric tests showed that the age and post-mortem interval are not associated with schizophrenia case/control phenotypes (*P*-value=0.17 and 0.09, respectively), and *χ*^2^-test showed that gender is not associated with case/control groups either (*P*-value=0.71). The sample description and RNA-seq data are available at https://www.ncbi.nlm.nih.gov/bioproject/379666.

### Library preparation and mRNA-seq

RNA-seq libraries were constructed using Illumina TruSeq RNA sample Prep Kit (Illumina, San Diego, CA, USA) following the manufacturer's instruction. The poly-A-containing mRNA molecules were purified from 300 to 500 ng DNAse-treated total RNA using oligo (dT) beads. Following the purification steps, the mRNA was fragmented into small pieces using divalent cations under elevated temperature (94 °C) for 2 min. Under these conditions, fragment lengths range from 130 to 290 bp with a median length of 185 bp. Reverse transcriptase and random primers were used to generate the first-strand complementary DNA from the cleaved RNA fragments. The second strand DNA was synthesized using DNA Polymerase I and RNaseH. These complementary DNA fragments then went through an end-repair process using T4 DNA polymerase, T4 PNK and Klenow DNA polymerase, and the addition of a single ‘A’ base using Klenow exo (3' to 5' exo minus), followed by ligation of the illumine PE adapters using T4 DNA Ligase. An index was inserted into illumina adapters so that multiple samples can be sequenced in a single lane. These products were then purified and enriched with polymerase chain reaction to create the final complementary DNA library for high-throughput DNA sequencing using Highseq2000 (Illumina). The concentration of RNA-seq libraries was measured by Qubit (Invitrogen, Waltham, MA, USA) and quantified with quantitative polymerase chain reaction. The quality of RNA-seq library was measured by LabChipGX (Caliper, MA, USA) using HT DNA 1 K/12 K/Hi-sensitivity LabChip. The libraries are multiplexed and loaded on a flowcell for cluster generation on cBot (Illumina). During sequencing, the Illumina Real Time Analysis module was used to perform image analysis and base calling, and the BCL Converter (CASAVA v1.8.2, Illumina) were followed to generate FASTQ files, which contain the sequence reads. Sequencing depth was over 80 million (2 × 100-bp 40 million paired-end) mappable sequencing reads.

### Sequencing data analysis

Genomic Short-read Nucleotide Alignment Program (version 2015-12-31.v9) was used to map the reads to the reference human genome (hg19). Common single-nucleotide polymorphisms (SNPs) recorded in dbSNP137 were taken into account to improve the alignment accuracy.^21^ The output SAM files were converted to BAM files, sorted by index. Duplication reads were removed using the SAMtools pipeline,^22^ and only unique, paired reads were used in the analysis. NCBI reference sequences (RefSeq) were used for gene annotation.^23^ Both fragments per kilobase of transcript per million mapped reads (FPKM)-based and count-based algorithms were used to identify the differentially expressed genes between cases and controls. The FPKM-based algorithm was implemented in the *Cuffdif*f package of Cufflinks2.2.1 (ref. 24) and two count-based algorithms were implemented in R packages edgeR^25^ and DEseq2.^26^ Default parameters of *Cuffdif*f, edgeR and DEseq2 were used for the gene differential expression analysis.

### Weighted gene co-expression network analysis

Co-expressed gene modules were detected by WGCNA. First, the normalized FPKM values of transcripts were calculated by the cuffnorm package of cufflinks.^24^ A matrix of correlations between all pairs of differentially expressed genes was generated by the FPKM values, and further converted into an adjacency matrix with a power function, so that the connection strength between two genes *x*_*i*_ and *x*_*j*_ was defined as *a*_*ij*_=|cor(*x*_*i*_, *x*_*j*_)|^*β*^. The parameter was determined by the criterion that the resulting adjacency matrix approximately fits a scale-free topological feature according to a model-fitting index proposed previously.^27^ The model-fitting index of a perfect scale-free network is 1. The value *β* of was 15, which is the minimum value required to make the model-fitting index above 0.8.^27^

The adjacency matrix was further transformed into a topological overlap matrix, which captures not only the direct interaction between two genes but also their indirect interactions through all the other genes in the network. A similarity measure was defined: TOM_*ij*_=(Σ_u_ *a*_*iu*_*a*_*uj*_+*a*_*ij*_)/(min(*k*_*i*_,*k*_*j*_)+1−*a*_*ij*_), where *k*_*i*_=Σ_u_ *a*_*iu*_ was the node connectivity.^28, 29^ 1−TOM_*ij*_ was used as a distance matrix in the hierarchical clustering of the transcript units for module detection.^28^

The module eigengene is the first principal component of the matrix of expression values of a given module, which was adopted to characterize the gene-expression profile of the module.

### Gene functional enrichment analysis

Gene functional enrichment analysis was applied on the differentially expressed genes and gene modules identified by WGCNA. DAVID (http://david.abcc.ncifcrf.gov/) was used to test enrichment in gene sets with gene ontology (GO) terms compared with the background list of all genes.^30^ Benjamini–Hochberg procedure was performed for multiple testing.

## Results

### Identification of differentially expressed genes

**Figure 1:**
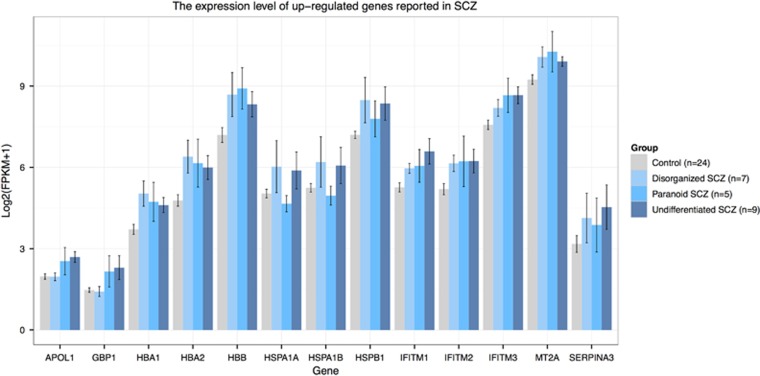
Bar plot showing expression levels of upregulated genes reported in previous schizophrenia transcriptome studies.

**Figure 2:**
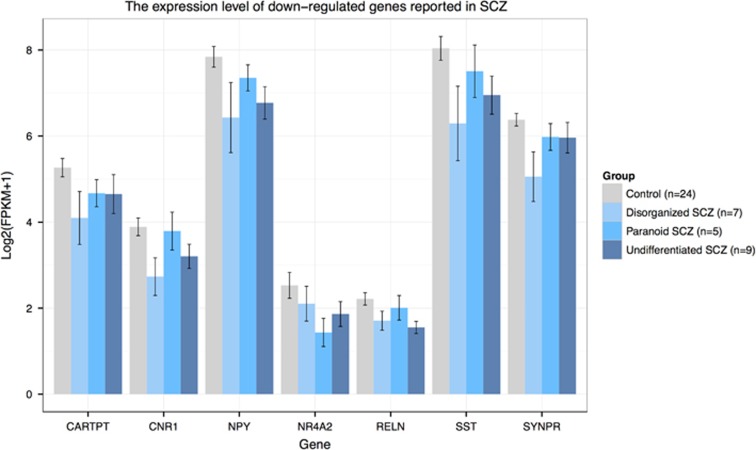
Bar plot showing expression levels of downregulated genes reported in previous schizophrenia transcriptome studies.

To search for differentially expressed genes, we employed multiple state-of-the-art statistical RNA-seq analysis workflows based on FPKM values and read counts,^31, 32^ built from the most mature and widely used computational tools, including *Cuffdif*f,^24^ edgeR^25^ and DEseq2.^26^ Cuffidiff, edgeR and DEseq2 identified 88, 99 and 104 differentially expressed genes, respectively, which have a *P*-value<0.05 after multiple testing corrections (Supplementary Figure S1 and Supplementary Table S2). Among those, 25 genes were identified by all three methods, including *HBA1*, *HBA2*, *HBB* and *IFITM1*, all of which have been reported to be differentially expressed in previous transcriptome studies in schizophrenia (Figures 1 and 2 and Supplementary Table S3).^15, 17, 33^ Several other and more highly differentially expressed genes showed significant *P*-values by one or two methods—all supported by previous studies. These include *GBP1*, *HSPA1A*, *HSPA1B*, *HSPB1*, *IFITM2*, *IFITM3*, *NPY*, *SST* and *SERPINA3* (Figures 1 and 2 and Supplementary Table S3).^9, 11, 12, 13, 15, 19^ In addition to the differentially expressed genes that met rigid statistical thresholds, multiple genes demonstrated nominal significance, several of which are involved in biological pathways of interest to the pathophysiology of schizophrenia. Further analysis of genes with a *P*-value<0.05, notwithstanding multiple testing corrections, included 761 genes shown to be of nominal significance by all three methods, and involved in downstream pathway and gene network functions of relevance to schizophrenia (Supplementary Figures S1 and 2 and Supplementary Table S4).

### Upregulation of gene pathways related to blood vessel development and immune response

Of the 761 (75%) genes analyzed, 569 were overexpressed in the amygdala of individuals with schizophrenia. Functional enrichment analysis of the 569 upregulated genes revealed that genes involved in 'blood vessel development' (GO: 0001568, *P*_corrected_=1.50 × 10^−^^7^), 'immune response' (GO: 0006955, *P*_corrected_=1.04 × 10^−4^), 'major histocompatibility complex (MHC) class I receptor activity' (GO: 0032393, *P*_corrected_=4.99 × 10^−3^) and 'inflammatory response' (GO: 0006954, *P*_corrected_=6.83 × 10^−3^) were significantly over-represented in cases compared with controls (Table 1). As the immune system activation has been repeatedly observed in many transcriptome studies of schizophrenia, we investigated whether the previously reported genes associated with immune systems were also dysregulated in amygdala. We found the expression of *SERPINA3*, *IFITM1*, *IFITM2* and *IFITM3*, *HSPA1A*, *HSPA1B*, *HSPB1*, *GBP1*, *MT2A* and *APOL1* to be upregulated in the amygdala of schizophrenia patients. Besides *SERPINA3*, two members of the serine proteinase inhibitor superfamily *SERPINE1* and *SERPING1* were also overexpressed. The paralogous genes of *GBP1*, *MT2A* and *APOL1*, including *GBP2*, *MT1G*, *MT1E*, *MT1F*, *MT1L* and *APOLD1,* were also upregulated in the schizophrenia cases (Supplementary Table S4).

### Suppression of gene pathways related to synaptic transmission and behavior

Of the 761 (25%) analyzed genes, 192 were downregulated in the amygdala of schizophrenia patients in comparison with controls. Functional enrichment analysis demonstrated that the 192 genes were significantly enriched in biological pathways including 'synaptic transmission' (GO: 0007268, *P*_corrected_=2.59 × 10^−5^), 'behavior' (GO: 0007610, *P*_corrected_=5.30 × 10^−5^), 'neuropeptide signaling pathway' (GO: 0007218, *P*_corrected_=4.62 × 10^−3^) and 'calcium ion-binding' (GO: 0005509, *P*_corrected_=7.89 × 10^−3;^Table 1). Notably, several GABAergic genes, which exhibited reduced expression in the prefrontal cortex of subjects with schizophrenia, were uncovered in our study, including *NPY*, *SST* and *RELN*.^34, 35, 36, 37^ Moreover, expression levels of genes involved in dopaminergic transmission, such as *CNR1*, *HTR2A*, *HTR2C, NR4A2*, *NTSR1*, *NXPH3* and *SLC17A8*, were also reduced (Supplementary Table S4).^38, 39, 40, 41, 42, 43^

### Comparison with previous RNA-seq studies in schizophrenia

We compared our results with two RNA-seq studies conducted on the prefrontal cortex and hippocampus of individuals with schizophrenia, respectively.^10, 15^ Fillman *et al.* identified 399 upregulated and 201 downregulated genes. Of those, 34 upregulated genes significantly overlapped with the upregulated genes in our study (*P*=2.82 × 10^−7^). Hwang *et al.* detected 123 upregulated and 21 downregulated genes. Among them, 50 upregulated genes significantly overlapped with the upregulated genes in our study (*P*=3.40 × 10^−42^). No downregulated genes overlapped between our study and any of the two previous studies. Gene functional enrichment analysis indicated that the upregulated genes identified by Fillman *et al.* were significantly enriched in 'inflammatory response' (GO: 0006954, *P*_corrected_=0.012) and 'blood vessel development' (GO: 0001568, *P*_corrected_=0.039), and nominally enriched in 'immune response' (GO: 0006955, *P*=6.13 × 10^−4^, *P*_corrected_=0.071; Supplementary Table S5). The upregulated genes identified by Hwang *et al.* were also nominally enriched in 'immune response' (GO: 0006955, *P*=1.06 × 10^−4^, *P*_corrected_=0.095; Supplementary Table S6). The enriched pathways of downregulated genes in our study were not significant in any of the two previous studies.

### Detection of functional modules in co-expression-based gene networks

**Figure 3:**
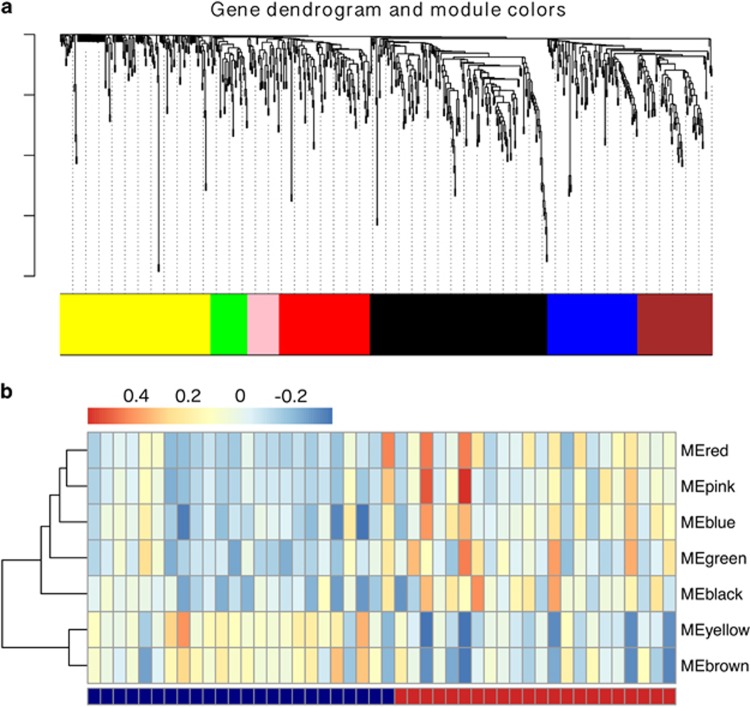
(**a**) The co-expression modules identified by weighted gene correlation network analysis (WGCNA). (**b**) The Eigengene heatmap of co-expression modules. Bottom legend: cases and controls are colored in red and blue, respectively. Top legend: Eigengene values of co-expression modules.

We further analyzed our data with WGCNA, which has been widely applied to study co-expression-based gene networks, both construction and module detection. Seven co-expression modules were identified including four modules that were significantly enriched in genes with specific pathway roles (see Figure 3 and Table 2: 'Module yellow', 176 genes, 'synaptic transmission' and 'calcium ion binding' 'Module green', 43 genes, 'blood vessel development' 'Module pink', 37 genes, 'immune response' 'Module red', 106 genes, 'immune response' and 'blood vessel development'). For each module, the eigengene was calculated to represent the overall expression profile of the genes in a certain module. The expression level of 'Module yellow' was low in schizophrenia cases, whereas expression levels of 'Module pink', 'Module red' and 'Module green' were high in cases, suggesting a high consistency between the pathway and co-expression-based gene network analysis.

A few highly connected nodes, which can hold the whole network together, represent the hubs of the network. Similarly, genes with a high number of interactions are considered to have an important role in organizing the biological network. Unlike protein–protein interaction networks, WGCNA defines a whole network connectivity measure (*k*_Total_) for each gene according to its Pearson correlation coefficient with all of the other genes, and an intramodular connectivity measure (*k*_Within_) when only considering the connection strength of each individual gene with all the other genes within the same module.

We further investigated whether the intramodular hub genes of a co-expression module were associated with the enriched pathways of the respective modules. Interestingly, two intramodular hubs of 'Module yellow' with the highest values of connectivity were GABA-related genes *NPY* and *SST*, and the top three connected intramodule hubs in 'Module green' were *ESAM*, *CDH5* and *MMRN2*, all of which are associated with angiogenesis or vascular diseases. The top intramodule hubs in 'Module pink' and 'Module red' were also strongly linked to the immune system (Supplementary Table S7).

## Discussion

Although multiple gene-expression studies have been performed on a variety of post-mortem brain tissues using both microarray and RNA-seq technologies, little attention has been devoted to examine transcriptome changes in the amygdala region of schizophrenia patients. To the best of our knowledge, no previous transcriptome study has specifically focused on subtypes of schizophrenia. Given the high degree of clinical heterogeneity of schizophrenia and the relatively small sample sizes of previous transcriptome studies (<50 samples), transcriptome changes in schizophrenia could be better understood by focusing on subgroups of patients with similar clinical manifestations.

In this study, we replicated many differentially expressed genes previously reported in schizophrenia, such as *IFITM1*, *IFITM2*, *IFITM3*, *NPY*, *SST* and *SERPINA3*. Elevated gene expression of the immune-related genes, including *IFITM1*, *IFITM2*, *IFITM3* and *SERPINA3*, has been repeatedly observed in the prefrontal cortex and hippocampus of patients with schizophrenia.^9, 10, 11, 13, 15^ Previous transcriptome studies have also revealed that genes involved in activation of the immune system are differentially expressed in schizophrenia, suggesting that they may contribute to the disease pathogenesis.^10, 11, 13, 15, 17, 18^ Here, we confirmed the previously observed overexpression of 'immune response' and 'inflammatory response' GO pathways in the amygdala region, using gene enrichment analysis. Our enrichment analysis also supported that upregulation of 'MHC class I receptor activity' results in keeping with the highly significant genome-wide association study association of the MHC region with schizophrenia. Although enrichment analysis of schizophrenia subtypes only showed upregulation of the 'immune response' pathway in undifferentiated and paranoid schizophrenia, we found that most differentially expressed genes involved in 'immune response', 'inflammatory response' and 'MHC class I receptor activity' pathways, including *IFITM1*, *IFITM2*, *IFITM3* and *SERPINA3*, are overexpressed in all three subtypes (Figure 1 and Supplementary Figures S3 and 4), suggesting that the activation of the immune system may be a common denominator in diverse subtypes of schizophrenia.

We additionally identified upregulated gene expression in the 'blood vessel development' pathway in schizophrenia. Further analyses of schizophrenia subtypes indicated that most differentially expressed genes involved in 'blood vessel development' are overexpressed in all three subtypes (Supplementary Figure S5). Enrichment analysis of schizophrenia subtypes also suggested elevated gene expression of the 'blood vessel development' pathway in all three subtypes. Moreover, we replicated the result by re-analyzing the differentially expressed genes from an independent RNA-seq study of the prefrontal cortex.^10^ The observation of altered gene expressions in immune system and angiogenesis lends supports to the vascular-inflammatory theory of schizophrenia. This theory hypothesized that inflammation involving the microvascular system leads to dysregulation of cerebral blood flow and damage to the blood–brain barrier, predisposing to the development of schizophrenia.^44^

Our study also confirmed that there is suppression of GABAgeric markers (that is, downregulation in *NPY*, *SST* and *RELN* expression) and showed that gene networks involving 'synaptic transmission', 'behavior', 'neuropeptide signaling pathway' and 'calcium ion-binding' pathways were also significantly downregulated in schizophrenia. Further analyses of schizophrenia subtypes indicated that most differentially expressed genes involved in 'synaptic transmission' and 'behavior' are downregulated in all three subtypes (Supplementary Figure S6). In contrast to the altered expression of immune-related gene pathways, the dysregulation of synaptic transmission or behavior pathway has not been previously reported in transcriptome-wide studies of schizophrenia.^9, 10, 11, 13, 14, 15, 17^ We also studied the differentially expressed gene sets of two published RNA-seq studies on the prefrontal cortex and hippocampus, respectively, in individuals with schizophrenia obeserving upregulation of immune pathways. Although speculative, the deficit of synaptic and behavioral genes in the amygdala may be more likely to associate with the behavioral phenotypes in schizophrenia, whereas the activation of the immune system may be associated with the more common phenotypes in schizophrenia.

We further investigated the differentially expressed genes among three subtypes of schizophrenia (Supplementary Note). Interestingly, our results showed that several genes previously implicated in schizophrenia are oppositely regulated in subtypes of schizophrenia. It is difficult to identify unified gene-expression profiles when analyzing a group of patients with diverse symptoms in schizophrenia. For example, genes involved in the neuregulin 1–ErbB4 signaling pathway, including *DOCK7*, *EGFR*, *PTPRZ1* and the key regulator *ERBB4*,^45, 46, 47^ were downregulated in undifferentiated and disorganized schizophrenia, while they were upregulated in paranoid schizophrenia (Supplementary Figure S7). Several genes that encode subunits of the NMDA receptor (*GRIN2D*), GABA receptor (*GABRA2* and *GABRB1*), GABA transporter (*SLC6A1* and *SLC6A11*) and glutamate transporter (*SLC1A3*) also displayed the same expression pattern. *NTRK2*, a susceptibility gene of paranoid schizophrenia,^48^ was not only overexpressed in paranoid schizophrenia, but also suppressed in undifferentiated and disorganized schizophrenia. In addition, the expression of *GRM3* was significantly reduced in undifferentiated schizophrenia, while it was increased in disorganized and paranoid schizophrenia (Supplementary Figure S7). Elevated expression of *CNTN2* and *EFHD1* was specifically found in disorganized schizophrenia (Supplementary Figure S7). In summary, the above gene-expression profiles appear to be associated with particular clinical phenotypes or subsets of schizophrenia patients, and could potentially serve as biomarkers to identify therapeutic targets for particular subtypes of schizophrenia.

We subsequently applied the unbiased WGCNA method on the differentially expressed genes to identify the key modules of highly co-expressed genes. Consistent with the results proposed above, four of seven detected modules were associated with immune response, vascular development or synaptic transmission, indicating that dysregulated genes that are involved in the same pathway are highly co-expressed. Further investigation of the intramodular hubs also confirmed that the genes that function as key hubs in a module have an important role in the enriched pathways of that module, such as *NPY*, *SST* in synaptic transmission, and *ESAM*, *CDH5* and *MMRN2* in blood vessel development, suggesting that the intramodular hub genes could function as potential biomarkers or be lead target sites for future therapeutic interventions. For example, modulators of *NPY* or *SST* signaling and drugs that increase the activity of the GABAergic pathway have been reviewed as attractive therapeutic opportunities for schizophrenia.^49, 50, 51^

In conclusion, we performed the first transcriptome study of amygdala in schizophrenia patients, using RNA-seq, and observed significant associations between specific transcriptome changes in amygdala and schizophrenia subtypes. Those include, but are not limited to, upregulation of genes that regulate activation of immune responses and angiogenesis pathways, and suppression of genes involved in synaptic transmission. Taken together, these gene networks may co-contribute in a significant way to the pathophysiology of schizophrenia. We conclude that our study provides new insights into the genomic underpinnings of schizophrenia and may allow for novel precision therapeutic development strategies targeting schizophrenia to be implemented in future studies.

## Figures and Tables

**Table 1:** Functional enrichment of differentially expressed genes

*Expression*	*Term*	*Fold enrichment*	P*-value*	*Benjamini*Q*-value*
Upregulation	GO:0001568~blood vessel development	4.06	6.06E−11	1.50E−07
	GO:0001944~vasculature development	3.96	1.13E−10	1.40E−07
	GO:0001525~angiogenesis	4.62	1.03E−08	8.46E−06
	GO:0048514~blood vessel morphogenesis	3.83	1.75E−08	1.08E−05
	GO:0009615~response to virus	5.14	6.40E−08	3.17E−05
	GO:0005576~extracellular region	1.66	1.08E−07	3.56E−05
	GO:0030198~extracellular matrix organization	5.08	1.90E−07	7.84E−05
	GO:0042127~regulation of cell proliferation	2.13	2.20E−07	7.78E−05
	GO:0044421~extracellular region part	2.04	2.29E−07	3.77E−05
	GO:0006955~immune response	2.21	3.36E−07	1.04E−04
	GO:0009611~response to wounding	2.29	2.95E−06	8.10E−04
	GO:0022610~biological adhesion	2.09	2.98E−06	7.37E−04
	GO:0051270~regulation of cell motion	3.38	3.95E−06	8.88E−04
	GO:0007155~cell adhesion	2.04	6.72E−06	1.28E−03
	GO:0032393~MHC class I receptor activity	13.23	7.98E−06	4.99E−03
	GO:0006952~defense response	2.02	3.79E−05	5.50E−03
	GO:0006954~inflammatory response	2.49	4.98E−05	6.83E−03
	GO:0019882~antigen processing and presentation	4.50	6.58E−05	8.10E−03
Downregulation	GO:0007268~synaptic transmission	5.94	2.33E−08	2.59E−05
	GO:0007610~behavior	4.44	9.56E−08	5.30E−05
	GO:0019226~transmission of nerve impulse	5.05	2.15E−07	7.94E−05
	GO:0006928~cell motion	3.72	1.15E−05	2.54E−03
	GO:0030534~adult behavior	9.68	1.75E−05	3.23E−03
	GO:0007218~neuropeptide signaling pathway	8.95	2.92E−05	4.62E−03
	GO:0005509~calcium ion binding	2.62	4.63E−05	7.89E−03
	GO:0051969~regulation of transmission of nerve impulse	6.37	8.08E−05	9.91E−03
	GO:0007409~axonogenesis	5.39	9.55E−05	1.05E−02
	GO:0031644~regulation of neurological system process	6.12	1.07E−04	1.07E−02
	GO:0050877~neurological system process	2.24	1.68E−04	1.54E−02
	GO:0048667~cell morphogenesis involved in neuron differentiation	4.98	1.75E−04	1.48E−02
	GO:0048812~neuron projection morphogenesis	4.89	2.01E−04	1.58E−02
	GO:0046873~metal ion transmembrane transporter activity	3.83	2.81E−04	3.15E−02
	GO:0005261~cation channel activity	4.19	2.82E−04	2.39E−02
	GO:0005216~ion channel activity	3.53	2.96E−04	2.01E−02
	GO:0050804~regulation of synaptic transmission	6.12	3.21E−04	2.34E−02
	GO:0007186~G-protein-coupled receptor protein signaling pathway	2.22	3.61E−04	2.47E−02

Abbreviations: GO, gene ontology; MHC, major histocompatibility complex.

**Table 2:** Functional enrichment of genes in co-expression modules.

*Module*	*Term*	*Fold enrichment*	P*-value*	*Benjamini*Q*-value*
Yellow	GO:0005576~extracellular region	2.56	3.59E−11	6.79E−09
	GO:0044421~extracellular region part	2.99	2.08E−07	1.97E−05
	GO:0007268~synaptic transmission	5.01	1.63E−06	2.21E−03
	GO:0010033~response to organic substance	3.17	2.51E−06	1.70E−03
	GO:0005509~calcium ion binding	2.85	7.29E−06	2.29E−03
	GO:0019226~transmission of nerve impulse	4.26	1.05E−05	4.72E−03
	GO:0005615~extracellular space	2.89	4.95E−05	3.12E−03
	GO:0007155~cell adhesion	2.84	6.54E−05	2.19E−02
	GO:0022610~biological adhesion	2.84	6.67E−05	1.79E−02
	GO:0016477~cell migration	4.32	9.98E−05	2.23E−02
Green	GO:0001568~blood vessel development	15.78	6.40E−09	3.65E−06
	GO:0001944~vasculature development	15.40	7.90E−09	2.25E−06
	GO:0048514~blood vessel morphogenesis	14.65	7.65E−07	1.45E−04
	GO:0001525~angiogenesis	15.67	3.16E−05	4.49E−03
	GO:0005886~plasma membrane	2.01	3.00E−04	3.13E−02
Pink	GO:0006955~immune response	6.76	6.38E−06	1.87E−03
Red	GO:0006955~immune response	3.84	2.92E−06	4.13E−03
	GO:0032403~protein complex binding	8.08	3.46E−06	8.55E−04
	GO:0002684~positive regulation of immune system process	6.80	4.34E−06	3.07E−03
	GO:0031349~positive regulation of defense response	14.10	9.30E−06	4.38E−03
	GO:0001775~cell activation	5.64	2.23E−05	7.88E−03
	GO:0006952~defense response	3.59	5.81E−05	1.64E−02
	GO:0030198~extracellular matrix organization	9.90	7.01E−05	1.64E−02
	GO:0005886~plasma membrane	1.67	1.51E−04	2.96E−02
	GO:0048584~positive regulation of response to stimulus	5.61	1.86E−04	3.70E−02
	GO:0045321~leukocyte activation	5.47	2.21E−04	3.84E−02
	GO:0007155~cell adhesion	3.15	2.28E−04	3.53E−02
	GO:0022610~biological adhesion	3.15	2.31E−04	3.23E−02
	GO:0001568~blood vessel development	5.40	2.40E−04	3.05E−02
	GO:0005911~cell−cell junction	6.33	2.48E−04	2.43E−02
	GO:0045089~positive regulation of innate immune response	15.98	2.52E−04	2.93E−02
	GO:0033256~I-kappaB/NF-kappaB complex	112.78	2.54E−04	1.67E−02
	GO:0001944~vasculature development	5.27	2.83E−04	3.04E−02
	GO:0046649~lymphocyte activation	5.91	3.82E−04	3.80E−02
	GO:0045088~regulation of innate immune response	13.62	4.69E−04	4.33E−02
	GO:0001525~angiogenesis	6.95	4.81E−04	4.17E−02
	GO:0046637~regulation of alpha−beta T-cell differentiation	23.53	6.08E−04	4.95E−02
	GO:0030098~lymphocyte differentiation	8.57	6.43E−04	4.94E−02

Abbreviations: GO, gene ontology; NF-kappaB, nuclear factor-κappaB.
