# Supplementary material for: RNA-seq analysis of amygdala tissue reveals characteristic expression profiles in schizophrenia
Source: Transl Psychiatry. 2017 Aug 15;7(8):e1203–. doi: 10.1038/tp.2017.154 (PMC5611723; doi:10.1038/tp.2017.154)
Supplement: Supplementary Information [file tp2017154x1.pdf]

Supplementary Note

Gene differential expression analysis by adjusting age and PMI

We also used DEseq2 to perform the DE gene analysis by including age and PMI as covariates. DEseq2 detected 2994 significant DE genes after multiple testing correction. Interestingly, this number is much bigger comparing to analysis without covariates. However, the presented genes such as *HBB*, *HBA2*, *HSPA1A*, *HSPA1B*, *HSPB1*, *IFITM1*, *GBP1*, *GBP2*, *CNR1*, *HTR2C*, *NPY*, *SST*, *RELN*, are still significant. We further performed function enrichment analysis on the significant genes, and found the results are highly consistent with our original analysis. Gene pathways, such as "blood vessel development" (GO: 0001568, $P_{corrected} = 2.09 \times 10^{-11}$), "regulation of immune system process" (GO: 0002682, $P_{corrected} = 0.013$) are significantly up-regulated, while "synaptic transmission" (GO: 0007268, $P_{corrected} = 1.64 \times 10^{-5}$), "behavior" (GO: 0007610, $P_{corrected} = 5.48 \times 10^{-12}$) are significantly down-regulated.

Gene differential expression analysis of 6 antipsychotics negative cases and 24 no-psychiatric controls

To exclude the potential effect of antipsychotics on gene expression, we analyzed the differentially expressed genes between 6 antipsychotics negative cases and controls. We identified 1109 DE genes including 910 up-regulated, and 199 down-regulated genes. Results of gene functional enrichment analysis are highly consistent with the original analysis. Up-regulated genes are significantly enriched in pathways such as "blood vessel development" (GO: 0001568, $P_{corrected} = 1.1 \times 10^{-5}$), "immune response" (GO: 0006955, $P_{corrected} = 3 \times 10^{-15}$), and "inflammatory response" (GO: 0006954, $P_{corrected} = 3 \times 10^{-10}$). Down-regulated genes are significantly enriched in pathways such as "neuropeptide signaling pathway" (GO: 0007218, $P_{corrected} = 1.4 \times 10^{-2}$).

Analysis of undifferentiated, disorganized, and paranoid subtypes of schizophrenia

We also calculated the differentially expressed genes between three schizophrenia subtypes (undifferentiated, disorganized and paranoid), and controls respectively. A total of 41 genes were significant after multiple testing corrections by all the three methods in undifferentiated schizophrenia, including *GBP1*, *HBA2*, *IFITM1*, *IFITM3*. In comparison, 12 genes were identified in disorganized schizophrenia including *HBA1* and *HBA2*. However, no gene was consistently significant after multiple testing corrections across different methods in paranoid schizophrenia, which may be due to the small number of cases.

Similarly, we also analyzed nominally significant genes identified by the three methods. This analysis revealed 798, 616 and 454 differentially expressed genes in the undifferentiated, disorganized and paranoid schizophrenia, respectively (**Table S8-10**). The similarity between the groups of differentially expressed genes was evaluated by Jaccard index, demonstrating that the overlap of differentially expressed genes was higher between undifferentiated and disorganized schizophrenia (**Table S11**).

Functional enrichment analysis of the 798 genes in undifferentiated schizophrenia indicated that the overexpressed genes are significantly enriched in "immune response" (GO: 0006955, $P_{corrected} = 5.18 \times 10^{-5}$) and "blood vessel development" (GO: 0001568, $P_{corrected} = 7.88 \times 10^{-5}$), while the suppressed genes are significantly enriched in behavior" (GO: 0007610, $P_{corrected} = 5.39 \times 10^{-5}$) and "synaptic transmission" (GO: 0007268, $P_{corrected} = 3.26 \times 10^{-3}$, **Table S12**). The down-regulated genes in disorganized schizophrenia are also significantly enriched in behavior" (GO: 0007610,

$P_{corrected}$ = 1.43 × 10^{-5}) and “synaptic transmission” (GO: 0007268, $P_{corrected}$ = 2.99 × 10^{-4}). Although no significantly enriched pathway was identified in the up-regulated genes in the disorganized schizophrenia samples, blood vessel development pathway was found to be nominally enriched (**Table S13**). In comparison, the up-regulated genes in patients with the paranoid schizophrenia subtype are significantly enriched in “blood vessel development” (GO: 0001568, $P_{corrected}$ = 1.22 × 10^{-3}) and “positive regulation of immune system process” (GO: 0002684, $P_{corrected}$ = 3.99 × 10^{-3}), while the down-regulated genes are significantly enriched in “nerve terminal” (GO: 0043476, $P_{corrected}$ = 3.20 × 10^{-4}) and axon (GO: 0030424, $P_{corrected}$ = 1.32 × 10^{-3}), and nominally enriched in fear response (GO: 0042596, P = 1.37 × 10^{-4}, $P_{corrected}$ = 0.116) and behavior (GO:0007610, P = 9.07 × 10^{-4}, $P_{corrected}$ = 0.151, **Table S14**).

Differentially expressed genes among subtypes of schizophrenia

We also analyzed the differentially expressed genes between each of the schizophrenia subtypes. No gene was significant after multiple testing corrections likely due to the small number of cases. However, 325, 293 and 98 nominally significant genes were identified from comparisons between undifferentiated-paranoid, disorganized-paranoid and undifferentiated-disorganized analyses, respectively (**Table S15-17**). Among them were genes such as *ERBB4*, *GRIN2D*, *GRM3* and *NTRK2* that have been previously shown to be associated with the pathogenesis of schizophrenia. Interestingly, *ERBB4*, *GRIN2D* and *NTRK2* are up-regulated in paranoid schizophrenia, while down-regulated in undifferentiated and disorganized schizophrenia. *GRM3* is up-regulated in disorganized and paranoid schizophrenia, while down-regulated in undifferentiated schizophrenia. *CNTN2* is highly up-regulated in disorganized schizophrenia, while down-regulated in paranoid schizophrenia (**Figure S7**).

Supplementary Tables

Table S1. Description of postmortem samples used in this study (included as a separate excel file).

Table S2. Significantly differentially expressed genes identified by Cuffdif, edgeR and DEseq2.

Cuffdif:

Gene	log2(fold_change)	*P*-value	Q-value
APOLD1	1.12	5.00E-05	0.01
ARHGAP36	-1.49	5.00E-05	0.01
ATOH8	0.91	5.00E-05	0.01
CEBPB	0.90	5.00E-05	0.01
CEBPD	1.00	5.00E-05	0.01
CLDN5	0.83	5.00E-05	0.01
CLIC1	0.87	5.00E-05	0.01
CRHBP	-1.03	5.00E-05	0.01
DNAJB1	2.50	5.00E-05	0.01
DOK7	1.45	5.00E-05	0.01
EDN1	1.19	5.00E-05	0.01
EMP1	1.52	5.00E-05	0.01
ENG	0.83	5.00E-05	0.01
FAM100B	0.68	5.00E-05	0.01
FGF17	1.13	5.00E-05	0.01
FSTL3	0.89	5.00E-05	0.01
G0S2	1.64	5.00E-05	0.01
GBP1	1.33	5.00E-05	0.01
GPR4	1.61	5.00E-05	0.01
HBA1	1.27	5.00E-05	0.01
HBA2	1.71	5.00E-05	0.01

HSPA1A	2.68	5.00E-05	0.01
HSPA1B	2.54	5.00E-05	0.01
HSPA6	6.76	5.00E-05	0.01
HSPB1	2.49	5.00E-05	0.01
ICAM1	1.81	5.00E-05	0.01
IFITM1	1.21	5.00E-05	0.01
ISLR2	-1.06	5.00E-05	0.01
ITGA5	1.07	5.00E-05	0.01
LOC100505495	1.65	5.00E-05	0.01
MAFF	1.76	5.00E-05	0.01
MKNK2	1.14	5.00E-05	0.01
MPZL2	1.71	5.00E-05	0.01
MSX1	1.24	5.00E-05	0.01
MT1G	1.05	5.00E-05	0.01
MTRNR2L10	1.37	5.00E-05	0.01
MTRNR2L5	1.09	5.00E-05	0.01
MTRNR2L7	1.15	5.00E-05	0.01
NPY	-0.90	5.00E-05	0.01
NR4A2	-1.36	5.00E-05	0.01
NXPH4	-1.12	5.00E-05	0.01
ODZ3	-0.97	5.00E-05	0.01
PCDH11X	-1.21	5.00E-05	0.01
PER1	0.84	5.00E-05	0.01
PPP1R15A	0.99	5.00E-05	0.01
RMRP	1.49	5.00E-05	0.01
SERPINH1	3.02	5.00E-05	0.01
SESN2	1.06	5.00E-05	0.01
SFRP2	-1.06	5.00E-05	0.01
SIX4	1.66	5.00E-05	0.01
SLC18A3	4.84	5.00E-05	0.01
SPOCD1	1.39	5.00E-05	0.01
TMC4	1.43	5.00E-05	0.01
TMEM155	-0.96	5.00E-05	0.01
TNFAIP2	0.87	5.00E-05	0.01
TNFRSF12A	2.08	5.00E-05	0.01
TPM2	0.81	5.00E-05	0.01
UNC5D	-1.12	5.00E-05	0.01
XLOC_010860	4.52	5.00E-05	0.01
ZCCHC12	-0.79	5.00E-05	0.01

BST2	0.92	1.00E-04	0.02
CFP	1.36	1.00E-04	0.02
FAM102B	-0.76	1.00E-04	0.02
HBB	1.53	1.00E-04	0.02
LHX8	2.97	1.00E-04	0.02
LOC338799	1.10	1.00E-04	0.02
MLKL	2.12	1.00E-04	0.02
RASIP1	0.90	1.00E-04	0.02
SCG2	-0.83	1.00E-04	0.02
SST	-0.90	1.00E-04	0.02
XLOC_011928	-1.47	1.00E-04	0.02
A4GALT	1.03	1.50E-04	0.03
C1R	0.81	1.50E-04	0.03
CNN2	1.15	1.50E-04	0.03
VWCE	0.92	1.50E-04	0.03
VWF	0.81	1.50E-04	0.03
XAF1	0.76	1.50E-04	0.03
ANXA2	0.84	2.00E-04	0.04
ATF3	1.24	2.00E-04	0.04
CD248	0.99	2.00E-04	0.04
CHI3L2	2.42	2.00E-04	0.04
IL4R	1.05	2.00E-04	0.04
LOC100216546	1.04	2.00E-04	0.04
LOC389332	-1.30	2.00E-04	0.04
NUPR1	0.73	2.00E-04	0.04
TBR1	-0.98	2.00E-04	0.04
CNR1	-0.73	2.50E-04	0.05
MIDN	0.65	2.50E-04	0.05

edgeR:

Gene	logFC	*P*-value	FDR
HSPA6	6.77	1.49E-14	1.88E-10
HSPA7	3.32	6.14E-10	3.88E-06
SERPINH1	3.04	2.81E-09	1.18E-05
HSPB1	2.52	5.01E-09	1.59E-05
DNAJB1	2.49	1.69E-08	4.28E-05
TNFRSF10D	2.44	7.04E-08	1.45E-04
HSPA1A	2.70	8.01E-08	1.45E-04
SFN	3.98	9.40E-08	1.49E-04

HSPA1B	2.56	1.74E-07	2.44E-04
TNFRSF12A	2.07	2.54E-07	3.21E-04
HBA2	1.75	7.63E-07	8.78E-04
TMC4	1.67	9.12E-07	9.62E-04
MLKL	1.98	2.10E-06	2.04E-03
ICAM1	1.83	2.76E-06	2.49E-03
HNRNPU-AS1	1.65	3.61E-06	3.04E-03
MTRNR2L1	1.48	5.26E-06	4.16E-03
MTRNR2L10	1.39	6.53E-06	4.86E-03
MTRNR2L3	1.44	7.52E-06	5.29E-03
BAG3	1.84	8.14E-06	5.42E-03
MTRNR2L6	1.40	8.98E-06	5.68E-03
MAB21L1	-1.46	1.43E-05	7.92E-03
FOSL1	2.68	1.47E-05	7.92E-03
ARHGAP36	-1.48	1.54E-05	7.92E-03
IFITM1	1.24	1.56E-05	7.92E-03
SCUBE1	-1.97	1.76E-05	8.45E-03
MKNK2	1.02	1.85E-05	8.45E-03
MTRNR2L9	1.26	1.90E-05	8.45E-03
RMRP	1.73	2.01E-05	8.45E-03
NGFR	2.17	2.07E-05	8.45E-03
SLC11A1	1.56	2.07E-05	8.45E-03
RN7SK	1.39	2.21E-05	8.75E-03
MSX1	1.25	2.32E-05	8.88E-03
HBA1	1.30	2.59E-05	9.64E-03
G0S2	1.62	3.06E-05	0.01
MT1H	2.12	3.18E-05	0.01
ODF3B	1.82	3.28E-05	0.01
IFI6	1.21	3.46E-05	0.01
HBB	1.59	3.71E-05	0.01
SERPINE1	1.74	3.96E-05	0.01
LINC01089	0.88	3.96E-05	0.01
SPOCD1	1.39	4.04E-05	0.01
GBP1	1.32	4.22E-05	0.01
SESN2	1.06	4.29E-05	0.01
MTRNR2L8	1.16	4.89E-05	0.01
LINC00324	1.65	5.12E-05	0.01
CEBPB	0.89	5.27E-05	0.01

SERPINA3	2.31	5.31E-05	0.01
EMP1	1.52	5.43E-05	0.01
CHI3L2	2.44	5.66E-05	0.01
FERMT3	1.33	5.75E-05	0.01
PER1	0.86	5.85E-05	0.01
ACRC	1.38	5.91E-05	0.01
PPP1R15A	1.00	6.31E-05	0.01
IFITM3	1.07	6.54E-05	0.01
CLEC2B	1.26	6.88E-05	0.02
BST2	0.88	7.12E-05	0.02
MTRNR2L5	1.15	7.17E-05	0.02
EDN1	1.19	8.10E-05	0.02
MTRNR2L2	1.10	8.12E-05	0.02
MAFF	1.69	1.06E-04	0.02
TNFAIP2	0.87	1.08E-04	0.02
MTRNR2L7	1.15	1.18E-04	0.02
FSTL3	0.84	1.31E-04	0.03
RSPO1	1.10	1.43E-04	0.03
CCDC81	1.48	1.45E-04	0.03
A4GALT	1.05	1.52E-04	0.03
GPR4	1.62	1.55E-04	0.03
BRE-AS1	1.61	1.58E-04	0.03
MC1R	1.10	1.64E-04	0.03
CRHBP	-1.02	1.78E-04	0.03
ATOH8	0.90	1.84E-04	0.03
PCAT19	1.28	1.90E-04	0.03
RASIP1	0.90	1.91E-04	0.03
CCDC114	1.81	2.01E-04	0.03
ENG	0.84	2.07E-04	0.03
GPR35	1.11	2.13E-04	0.03
CNN2	1.17	2.14E-04	0.03
IFITM2	1.31	2.19E-04	0.03
RPPH1	1.36	2.20E-04	0.03
CD248	0.98	2.20E-04	0.03
IL1RL1	3.81	2.27E-04	0.03
SQRDL	0.79	2.41E-04	0.04
SLC52A3	1.16	2.44E-04	0.04
ZNF358	0.54	2.48E-04	0.04

PRKD2	0.82	2.57E-04	0.04
EPHA2	1.17	2.59E-04	0.04
NXPH4	-1.10	2.78E-04	0.04
TYMP	1.39	2.80E-04	0.04
GBP2	1.07	3.01E-04	0.04
SOX18	0.99	3.21E-04	0.04
UBALD2	0.69	3.24E-04	0.04
IL32	1.21	3.36E-04	0.04
PLIN2	0.72	3.66E-04	0.05
UPP1	0.65	3.68E-04	0.05
XAF1	0.74	3.76E-04	0.05
PCDH11X	-1.18	3.77E-04	0.05
TUBA1C	0.87	4.01E-04	0.05
TPM2	0.85	4.04E-04	0.05
GGT5	0.81	4.04E-04	0.05

DEseq2:

**Gene**	**log2(fold_change)**	***P*-value**	**FDR**
SLCO4A1	1.53	1.76E-11	3.42E-07
TUBB6	1.13	1.81E-10	1.76E-06
YBX3	1.28	2.06E-09	1.33E-05
NNMT	1.10	1.24E-08	6.04E-05
C2CD4B	1.29	7.90E-08	3.07E-04
BARHL2	-1.28	1.13E-07	3.67E-04
TNFRSF12A	1.22	2.78E-07	7.20E-04
GDF15	1.22	2.96E-07	7.20E-04
S100A8	1.17	1.15E-06	2.49E-03
KIAA0040	0.93	1.51E-06	2.66E-03
HBA2	1.11	1.42E-06	2.66E-03
FAM167B	1.08	2.13E-06	3.18E-03
HLX	0.89	2.47E-06	3.18E-03
LAG3	1.08	2.54E-06	3.18E-03
MT1A	1.06	2.60E-06	3.18E-03
TNFRSF6B	0.91	2.61E-06	3.18E-03
ZC3H12A	1.07	2.95E-06	3.38E-03
ICAM1	1.08	4.61E-06	4.98E-03
CD164L2	1.08	8.15E-06	6.60E-03
MAB21L1	-1.00	8.12E-06	6.60E-03

SLC5A10	0.98	7.98E-06	6.60E-03
ITPKC	0.79	7.69E-06	6.60E-03
TNFRSF10D	1.06	7.84E-06	6.60E-03
CFP	0.99	6.61E-06	6.60E-03
MTRNR2L1	1.00	8.61E-06	6.69E-03
MTRNR2L10	0.97	1.06E-05	7.92E-03
MTRNR2L3	0.99	1.12E-05	8.09E-03
MPZL2	1.02	1.36E-05	9.45E-03
MTRNR2L6	0.97	1.43E-05	9.60E-03
GPR97	1.01	1.62E-05	0.01
ARHGAP36	-0.98	1.70E-05	0.01
DOK7	0.89	2.00E-05	0.01
PAPPA2	-0.98	2.43E-05	0.01
LOC284454	0.92	2.46E-05	0.01
MSX1	0.91	2.45E-05	0.01
PPP1R17	-0.95	2.53E-05	0.01
IFITM1	0.89	3.19E-05	0.01
HBA1	0.91	3.21E-05	0.01
JSRP1	0.98	3.06E-05	0.01
SLC11A1	0.97	2.93E-05	0.01
OSMR	0.89	3.28E-05	0.01
SLC44A4	0.99	3.21E-05	0.01
MTRNR2L9	0.90	3.05E-05	0.01
MKNK2	0.81	3.58E-05	0.01
IL18R1	0.96	3.54E-05	0.01
RN7SK	0.94	3.57E-05	0.01
LCN1	1.00	3.49E-05	0.01
C11orf91	0.95	3.71E-05	0.01
MUC5B	0.94	4.06E-05	0.02
DLX4	0.97	5.30E-05	0.02
LINC01089	0.73	6.05E-05	0.02
HBB	0.95	6.16E-05	0.02
CLEC2B	0.88	6.57E-05	0.02
IRS4	-0.95	7.63E-05	0.03
MTRNR2L8	0.84	7.89E-05	0.03
CEBPB	0.73	8.46E-05	0.03
MTRNR2L5	0.83	9.97E-05	0.03
EMP1	0.92	9.98E-05	0.03

UPK1A	0.94	9.90E-05	0.03
EDN1	0.84	9.78E-05	0.03
STEAP4	0.85	9.79E-05	0.03
LRG1	0.94	1.01E-04	0.03
LOC728715	0.81	1.04E-04	0.03
PER1	0.71	1.10E-04	0.03
DES	0.88	1.12E-04	0.03
IFITM3	0.80	1.22E-04	0.03
BST2	0.72	1.22E-04	0.03
MTRNR2L2	0.81	1.22E-04	0.03
CPA4	0.91	1.18E-04	0.03
SQRDL	0.66	1.41E-04	0.04
NFKBIZ	0.72	1.44E-04	0.04
HPD	0.85	1.48E-04	0.04
OSM	0.90	1.51E-04	0.04
A4GALT	0.78	1.50E-04	0.04
MAFF	0.91	1.54E-04	0.04
C19orf35	0.87	1.63E-04	0.04
MTRNR2L7	0.82	1.75E-04	0.04
AURKC	0.75	1.77E-04	0.04
CD248	0.75	2.02E-04	0.04
TNFAIP2	0.70	1.98E-04	0.04
NPIPB6	0.86	2.01E-04	0.04
FSTL3	0.69	2.00E-04	0.04
GPR4	0.89	1.96E-04	0.04
GPR35	0.80	2.08E-04	0.04
FUT7	0.84	2.08E-04	0.04
CNN2	0.82	2.23E-04	0.05
EPHA2	0.82	2.34E-04	0.05
ZNF358	0.49	2.42E-04	0.05
PCAT19	0.84	2.30E-04	0.05
LAIR2	-0.88	2.44E-04	0.05
ATOH8	0.72	2.30E-04	0.05
SLC52A3	0.81	2.43E-04	0.05
FER1L4	0.72	2.39E-04	0.05
EOGT	0.43	2.36E-04	0.05
MSLNL	0.88	2.47E-04	0.05
RSPO1	0.79	2.66E-04	0.05

DRD4	0.63	2.61E-04	0.05
HBD	0.88	2.58E-04	0.05
EXPH5	0.64	2.73E-04	0.05
GZMM	0.82	2.60E-04	0.05
MISP	0.77	2.63E-04	0.05
GNRH2	0.73	2.73E-04	0.05
ARAP3	0.55	2.69E-04	0.05
PARP12	0.59	2.57E-04	0.05

Table S3. Dysregulated genes reported by previous schizophrenia transcriptome studies, which were differentially expressed in our study.

Gene	Significance	Direction	RNA-seq	Microarray	Other
APOL1	++	Up	Hwang et al, 2013	Mimmack et al, 2002	
GBP1	++	Up		Saetre, 2007	
HBA1	+++	Up	Xu et al, 2012; Hwang et al, 2013		
HBA2	+++	Up	Xu et al, 2012; Hwang et al, 2013		
HBB	+++	Up	Xu et al, 2012; Hwang et al, 2013; Fillman et al, 2013		
HSPA1A	+	Up		Arion et al, 2007	
HSPA1B	+	Up		Arion et al, 2007	
HSPB1	++	Up	Fillman et al, 2013	Arion et al, 2007	
IFITM1	+++	Up	Hwang et al, 2013		
IFITM2	++	Up	Wu et al, 2012; Hwang et al, 2013; Fillman et al, 2013	Arion et al, 2007; Saetre et al, 2007	
IFITM3	++	Up	Wu et al, 2012; Hwang et al, 2013	Arion et al, 2007; Saetre et al, 2007	
MT2A	++	Up	Wu et al, 2012	Arion et al, 2007; Choi et al, 2008; Logotheti, 2012	
SERPINA3	++	Up	Fillman et al, 2013	Arion et al, 2007; Saetre et al, 2007	
CARTPT	++	Down			Guillozet-Bongaarts et al, 2014
CNR1	++	Down			Guillozet-Bongaarts et al, 2014
NPY	++	Down		Weidenhofer et al, 2006; Choi et al, 2008; Hashimoto et al, 2008	Guillozet-Bongaarts et al, 2014; Mellios et al, 2009;
NR4A2	++	Down		Choi et al, 2008	Guillozet-Bongaarts et al, 2014
RELN	++	Down			Guodotti et al, 2000; Fatemi et al, 2004
SST	++	Down		Choi et al, 2008; Hashimoto et al, 2008	Guillozet-Bongaarts et al, 2014
SYNPR	++	Down			Guillozet-Bongaarts et al, 2014

"+++": significant by all the three methods after multiple testing.
"++": significant by all the three methods without multiple testing.
"+":significant by at least one method after multiple testing.
Reference: (1-13).

Table S4. 761 genes showed *P*-value < 0.05 (without multiple testing correction) by all three methods in the analysis of 22 schizophrenia cases and 24 controls (included as a separate excel file).

Table S5. Functional enrichment of differentially expressed genes reported by Fillman et al (9).

Expression	Term	Fold Enrichment	*P*-value	Benjamini *Q*-value
Up-regulation	GO:0010033~response to organic substance	2.79	1.54E-07	2.97E-04
	GO:0009611~response to wounding	3.01	9.92E-07	9.57E-04
	GO:0009266~response to temperature stimulus	7.83	1.26E-06	8.10E-04
	GO:0006952~defense response	2.69	5.01E-06	2.42E-03
	GO:0005615~extracellular space	2.52	1.07E-05	2.45E-03
	GO:0044421~extracellular region part	2.17	2.40E-05	2.74E-03
	GO:0006986~response to unfolded protein	7.49	2.46E-05	9.44E-03
	GO:0006954~inflammatory response	3.27	3.67E-05	1.17E-02
	GO:0009408~response to heat	8.44	4.05E-05	1.11E-02
	GO:0051789~response to protein stimulus	5.52	8.02E-05	1.92E-02
	GO:0045765~regulation of angiogenesis	7.50	8.74E-05	1.86E-02
	GO:0009991~response to extracellular stimulus	3.76	9.18E-05	1.76E-02
	GO:0048545~response to steroid hormone stimulus	4.00	1.01E-04	1.75E-02
	GO:0009628~response to abiotic stimulus	2.89	1.69E-04	2.68E-02
	GO:0001568~blood vessel development	3.38	2.66E-04	3.88E-02
	GO:0005886~plasma membrane	1.39	3.09E-04	2.33E-02
	GO:0001944~vasculature development	3.29	3.37E-04	4.54E-02
	GO:0005576~extracellular region	1.60	3.71E-04	2.10E-02
	GO:0000187~activation of MAPK activity	5.76	4.58E-04	5.73E-02
	GO:0006955~immune response	2.14	6.13E-04	7.14E-02
Down-regulation	GO:0005576~extracellular region	2.07	1.02E-05	1.54E-03
	GO:0044421~extracellular region part	2.62	4.46E-05	3.38E-03
	GO:0005578~proteinaceous extracellular matrix	3.76	6.57E-04	3.27E-02
	GO:0031012~extracellular matrix	3.48	1.17E-03	4.34E-02

Table S6. Functional enrichment of differentially expressed genes reported by Hwang et al (10).

Expression	Term	Fold Enrichment	*P*-value	Benjamini *Q*-value
Up-regulation	GO:0005576~extracellular region	2.31	1.35E-05	2.31E-03
	GO:0006955~immune response	3.38	1.06E-04	9.50E-02
	GO:0044421~extracellular region part	2.83	2.16E-04	1.84E-02
	GO:0005615~extracellular space	3.03	9.58E-04	5.35E-02

Table S7. Connectivity strength of intra-hub genes in the functional enriched modules. For module yellow and red (>100 genes), the top 10 connected genes (k_{within}) were listed. For module green and pink (<50 genes), the top 5 connected genes (k_{within}) were listed.

Module	Gene	k_{Total}	k_{Within}	k_{Out}	k_{Diff}
yellow (n=176)	*NPY*	1.18	0.80	0.38	0.41
	SST	1.14	0.76	0.38	0.39
	SCG2	1.07	0.62	0.44	0.18
	TAGLN	0.75	0.62	0.13	0.49
	CORT	0.90	0.59	0.32	0.27
	APBB1IP	0.54	0.54	0.00	0.54
	MYL9	0.55	0.51	0.04	0.48
	PLD4	0.51	0.51	0.00	0.51
	SORCS3	1.49	0.47	1.02	-0.55
	SYNPR	0.76	0.43	0.33	0.10
red (n=106)	*TRIP10*	10.61	6.21	4.40	1.81
	RELA	9.69	5.49	4.20	1.29
	PXN	9.51	4.94	4.58	0.36
	IL4R	4.99	4.03	0.96	3.07
	NFKB2	5.60	3.94	1.66	2.29
	LRRC32	6.18	3.77	2.41	1.36
	CLIC1	5.52	3.73	1.79	1.94
	ICAM2	6.06	3.72	2.34	1.38
	MAFF	4.10	3.54	0.56	2.97
	IFITM2	7.14	3.50	3.65	-0.15
pink (n=37)	*TAP1*	5.04	1.28	3.76	-2.48
	NLRC5	2.12	1.20	0.92	0.29
	GBP1	1.47	1.08	0.38	0.70
	C1R	2.36	1.03	1.33	-0.30
	DTX3L	1.80	0.94	0.86	0.08
green (n=43)	*ESAM*	4.53	2.60	1.93	0.67
	CDH5	4.12	2.27	1.84	0.43
	MMRN2	3.11	2.00	1.11	0.89
	ENG	3.66	1.88	1.78	0.10
	ROBO4	2.64	1.70	0.94	0.77

Table S8. 798 genes showed *P*-value < 0.05 (without multiple testing correction) by all three methods in the analysis of 9 undifferentiated schizophrenia cases and 24 controls (included as a separate excel file).

Table S9. 616 genes showed *P*-value < 0.05 (without multiple testing correction) by all three methods in the analysis of 7 disorganized schizophrenia cases and 24 controls (included as a separate excel file).

Table S10. 454 genes showed *P*-value < 0.05 (without multiple testing correction) by all three methods in the analysis of 5 paranoid schizophrenia cases and 24 controls (included as a separate excel file).

Table S11. Jaccard index between undifferentiated, disorganized and paranoid schizophrenia.

Jaccard index	Undifferentiated	Disorganized	Paranoid
**Undifferentiated**		0.1744	0.0821
**Disorganized**			0.0563
**Paranoid**			

Table S12. Functional enrichment of differentially expressed genes in differentiated schizophrenia.

Expression	Term	Fold Enrichment	P-value	Benjamini Q-value
Up-regulation	GO:0006955~immune response	2.13	1.94E-08	5.18E-05
	GO:0001568~blood vessel development	3.05	5.89E-08	7.88E-05
	GO:0001525~angiogenesis	3.79	7.28E-08	6.49E-05
	GO:0001944~vasculature development	2.98	1.02E-07	6.84E-05
	GO:0006952~defense response	2.09	3.56E-07	1.90E-04
	GO:0048514~blood vessel morphogenesis	2.88	3.78E-06	1.44E-03
	GO:0009611~response to wounding	1.98	1.89E-05	6.29E-03
	GO:0006954~inflammatory response	2.30	2.48E-05	7.34E-03
	GO:0045087~innate immune response	3.05	8.16E-05	2.16E-02
Down-regulation	GO:0007610~behavior	3.57	3.47E-08	5.39E-05
	GO:0043005~neuron projection	3.46	2.83E-06	7.00E-04
	GO:0019226~transmission of nerve impulse	3.55	3.65E-06	2.84E-03
	GO:0007268~synaptic transmission	3.75	6.30E-06	3.26E-03
	GO:0006928~cell motion	3.00	8.02E-06	3.11E-03
	GO:0048015~phosphoinositide-mediated signaling	7.05	1.14E-05	3.52E-03
	GO:0030001~metal ion transport	2.94	1.91E-05	4.94E-03
	GO:0031420~alkali metal ion binding	3.99	2.39E-05	1.15E-02
	GO:0030182~neuron differentiation	2.98	2.60E-05	5.75E-03
	GO:0044456~synapse part	3.66	3.17E-05	3.92E-03
	GO:0021953~central nervous system neuron differentiation	10.86	3.87E-05	7.49E-03
	GO:0016477~cell migration	3.60	3.92E-05	6.74E-03
	GO:0050804~regulation of synaptic transmission	5.02	6.71E-05	1.04E-02
	GO:0007611~learning or memory	5.59	7.31E-05	1.03E-02
	GO:0051969~regulation of transmission of nerve impulse	4.64	1.29E-04	1.65E-02
	GO:0048870~cell motility	3.23	1.31E-04	1.55E-02
	GO:0051674~localization of cell	3.23	1.31E-04	1.55E-02
	GO:0016021~integral to membrane	1.30	1.43E-04	1.18E-02
	GO:0021954~central nervous system neuron development	11.64	1.43E-04	1.58E-02
	GO:0044459~plasma membrane part	1.58	1.44E-04	8.90E-03
	GO:0031224~intrinsic to membrane	1.28	1.75E-04	8.66E-03
	GO:0031644~regulation of neurological system process	4.46	1.79E-04	1.84E-02
	GO:0015672~monovalent inorganic cation transport	3.12	1.92E-04	1.85E-02
	GO:0045202~synapse	2.86	1.93E-04	7.96E-03
	GO:0006812~cation transport	2.47	2.24E-04	2.03E-02

	GO:0040012~regulation of locomotion	3.88	2.71E-04	2.32E-02
	GO:0048666~neuron development	2.93	3.80E-04	3.06E-02
	GO:0008021~synaptic vesicle	5.93	3.82E-04	1.35E-02
	GO:0019932~second-messenger-mediated signaling	3.43	4.12E-04	3.15E-02
	GO:0030136~clathrin-coated vesicle	4.27	5.56E-04	1.71E-02
	GO:0034702~ion channel complex	3.30	1.06E-03	2.87E-02
	GO:0007409~axonogenesis	3.54	1.12E-03	6.46E-02
	GO:0030424~axon	3.54	2.07E-03	3.87E-02
	GO:0030135~coated vesicle	3.54	2.07E-03	3.87E-02

Table S13. Functional enrichment of differentially expressed genes in disorganized schizophrenia.

Expression	Term	Fold Enrichment	P-value	Benjamini Q-value
Up-regulation	GO:0001525~angiogenesis	3.99	2.12E-04	3.01E-01
	GO:0001568~blood vessel development	3.01	4.78E-04	2.36E-01
	GO:0001944~vasculature development	2.94	6.09E-04	2.27E-01
	GO:0016460~myosin II complex	12.26	6.38E-04	1.66E-01
	GO:0009925~basal plasma membrane	10.37	1.23E-03	1.60E-01
	GO:0016459~myosin complex	5.81	1.24E-03	1.11E-01
	GO:0007517~muscle organ development	3.03	1.25E-03	2.96E-01
	GO:0048514~blood vessel morphogenesis	3.03	1.25E-03	2.96E-01
Down-regulation	GO:0007610~behavior	4.22	9.83E-09	1.43E-05
	GO:0015267~channel activity	4.23	4.73E-08	2.05E-05
	GO:0022836~gated channel activity	4.85	6.63E-08	9.57E-06
	GO:0005216~ion channel activity	4.31	7.76E-08	8.40E-06
	GO:0045202~synapse	4.13	1.60E-07	9.37E-06
	GO:0019226~transmission of nerve impulse	4.48	2.24E-07	1.62E-04
	GO:0031226~intrinsic to plasma membrane	2.36	3.37E-07	1.32E-05
	GO:0007268~synaptic transmission	4.71	6.19E-07	2.99E-04
	GO:0044057~regulation of system process	4.54	1.00E-06	3.63E-04
	GO:0008021~synaptic vesicle	9.19	1.24E-06	4.14E-05
	GO:0043005~neuron projection	3.88	1.84E-06	4.31E-05
	GO:0005261~cation channel activity	4.61	1.92E-06	1.39E-04
	GO:0007267~cell-cell signaling	3.02	1.08E-05	2.59E-03
	GO:0007155~cell adhesion	2.83	1.10E-05	2.28E-03
	GO:0051969~regulation of transmission of nerve impulse	6.17	1.13E-05	2.04E-03
	GO:0022610~biological adhesion	2.82	1.13E-05	1.82E-03
	GO:0031644~regulation of neurological system process	5.93	1.60E-05	2.32E-03
	GO:0050804~regulation of synaptic transmission	6.07	3.89E-05	4.02E-03
	GO:0007611~learning or memory	6.69	5.77E-05	5.56E-03
	GO:0030424~axon	4.83	9.14E-05	1.43E-03
	GO:0007409~axonogenesis	4.70	1.15E-04	1.04E-02
	GO:0030594~neurotransmitter receptor activity	6.67	1.89E-04	6.28E-03
	GO:0048666~neuron development	3.41	2.35E-04	1.61E-02
	GO:0048812~neuron projection morphogenesis	4.26	2.57E-04	1.68E-02
	GO:0031175~neuron projection development	3.87	2.73E-04	1.71E-02
	GO:0007166~cell surface receptor linked signal	1.78	2.95E-04	1.77E-02

	transduction			
	GO:0007411~axon guidance	6.17	3.08E-04	1.77E-02
	GO:0042165~neurotransmitter binding	6.15	3.12E-04	9.60E-03

Table S14. Functional enrichment of differentially expressed genes in paranoid schizophrenia.

Expression	Term	Fold Enrichment	P-value	Benjamini Q-value
Up-regulation	GO:0031012~extracellular matrix	3.54	8.34E-08	1.17E-05
	GO:0005576~extracellular region	1.80	1.40E-07	1.30E-05
	GO:0005578~proteinaceous extracellular matrix	3.38	1.26E-06	8.81E-05
	GO:0031226~intrinsic to plasma membrane	1.97	3.32E-06	1.86E-04
	GO:0005887~integral to plasma membrane	1.98	3.99E-06	1.86E-04
	GO:0051270~regulation of cell motion	4.03	5.07E-06	3.83E-03
	GO:0008284~positive regulation of cell proliferation	2.76	1.32E-05	7.44E-03
	GO:0009986~cell surface	2.97	1.66E-05	6.65E-04
	GO:0001568~blood vessel development	3.36	2.71E-05	1.22E-02
	GO:0001944~vasculature development	3.28	3.68E-05	1.38E-02
	GO:0009611~response to wounding	2.41	3.77E-05	1.21E-02
	GO:0042127~regulation of cell proliferation	2.03	1.07E-04	2.65E-02
	GO:0030335~positive regulation of cell migration	5.14	1.36E-04	3.04E-02
	GO:0032101~regulation of response to external stimulus	3.74	1.90E-04	3.84E-02
	GO:0048514~blood vessel morphogenesis	3.25	2.21E-04	4.09E-02
	GO:0002684~positive regulation of immune system process	3.07	2.33E-04	3.99E-02
	GO:0006955~immune response	1.92	1.17E-03	1.44E-01
Down-regulation	GO:0043679~nerve terminal	28.01	2.00E-06	3.20E-04
	GO:0005576~extracellular region	2.32	2.46E-05	1.31E-03
	GO:0030424~axon	8.69	3.31E-05	1.32E-03
	GO:0043005~neuron projection	5.05	1.36E-04	3.62E-03
	GO:0042596~fear response	38.49	1.37E-04	1.16E-01
	GO:0042995~cell projection	3.47	1.40E-04	3.19E-03
	GO:0010817~regulation of hormone levels	8.47	1.61E-04	6.99E-02
	GO:0009991~response to extracellular stimulus	6.65	1.79E-04	5.24E-02
	GO:0007194~negative regulation of adenylate cyclase activity	16.62	2.16E-04	4.75E-02
	GO:0051350~negative regulation of lyase activity	16.62	2.16E-04	4.75E-02
	GO:0031280~negative regulation of cyclase activity	16.62	2.16E-04	4.75E-02
	GO:0007610~behavior	3.90	9.07E-04	1.51E-01
	GO:0030165~PDZ domain binding	15.78	1.97E-03	3.58E-01

Table S15. 325 genes showed *P*-value < 0.05 (without multiple testing correction) by all three methods in the analysis of 9 undifferentiated schizophrenia and 5 paranoid schizophrenia patients (included as a separate excel file).

Table S16. 293 genes showed *P*-value < 0.05 (without multiple testing correction) by all three methods in the analysis of 7 disorganized schizophrenia and 5 paranoid schizophrenia patients (included as a separate excel file).

Table S17. 98 genes showed *P*-value < 0.05 (without multiple testing correction) by all three methods in the analysis of 9 undifferentiated schizophrenia and 7 disorganized schizophrenia patients (included as a separate excel file).

Supplementary Figures

Figure S1. Venn diagram of differentially expressed genes among three methods.
a. significant genes after multiple testing correction.

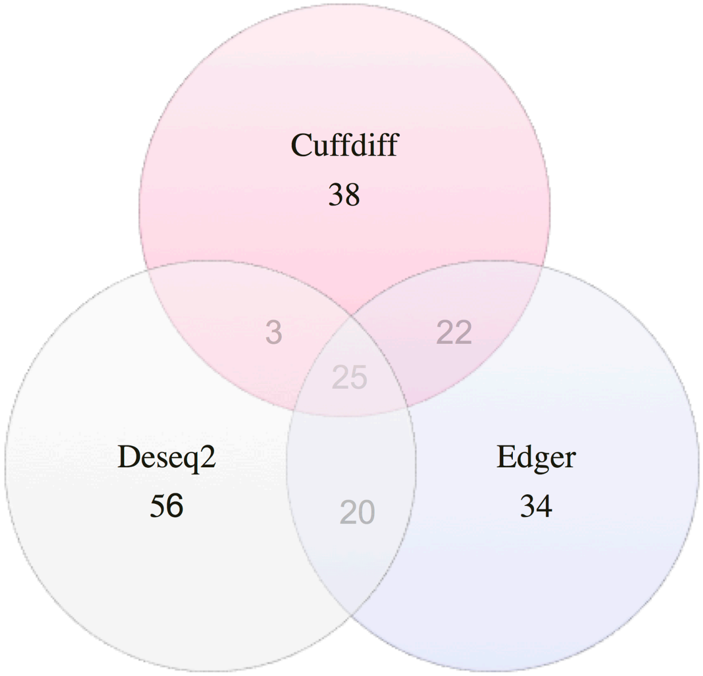

b. significant genes without multiple testing correction.

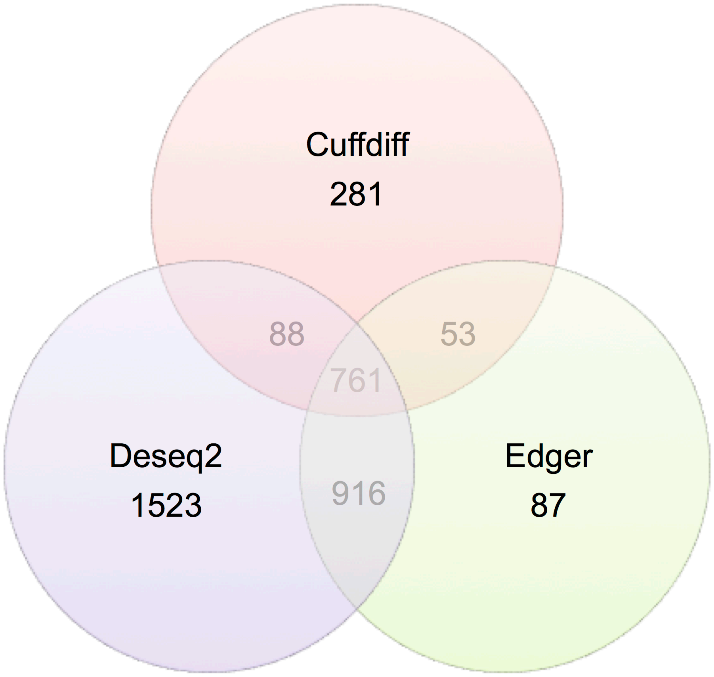

Figure S2. Heatmap shows expression levels of 761 differentially expressed genes (without multiple testing correction) identified by three methods. Left legend: cases and controls are colored in red and blue respectively. Right legend: Standardized Z-scores based on the FPKM value.

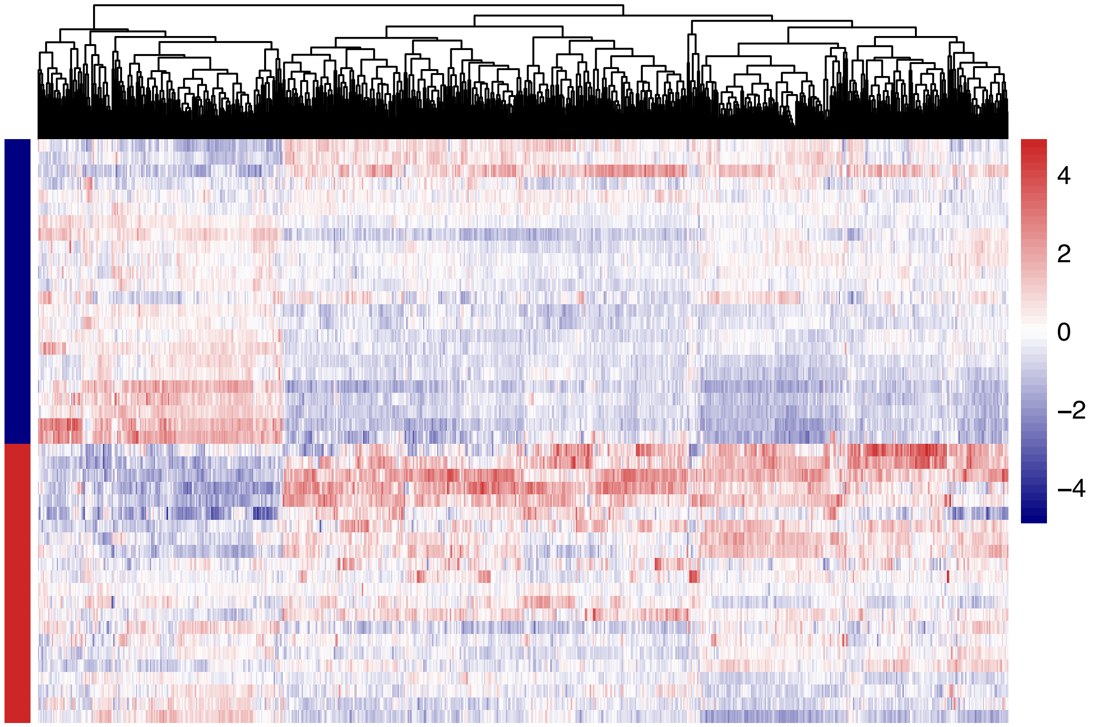

Figure S3. Barplot showing expression levels of up-regulated genes in MHC class I receptor activity.

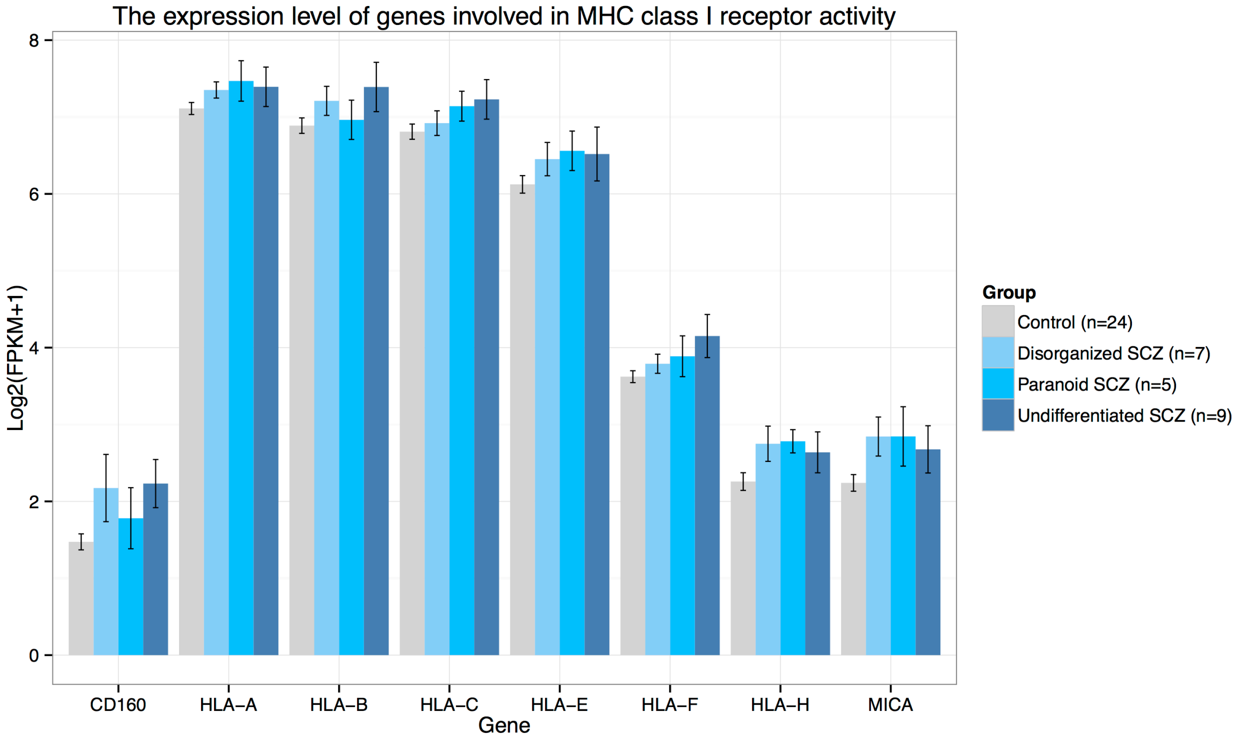

Figure S4. Barplot showing expression levels of up-regulated genes in immune and inflammatory response.

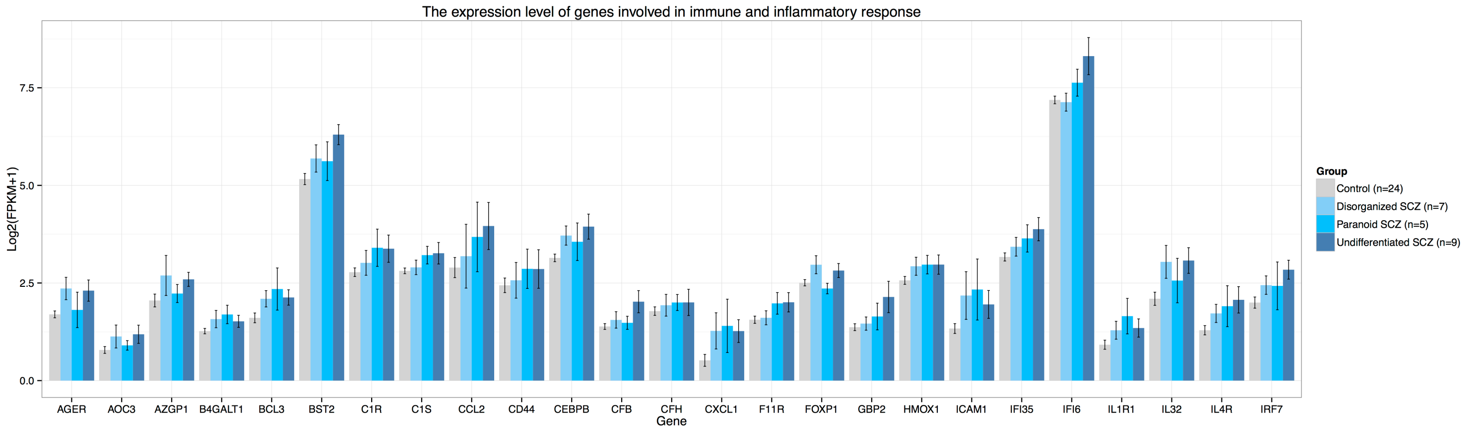

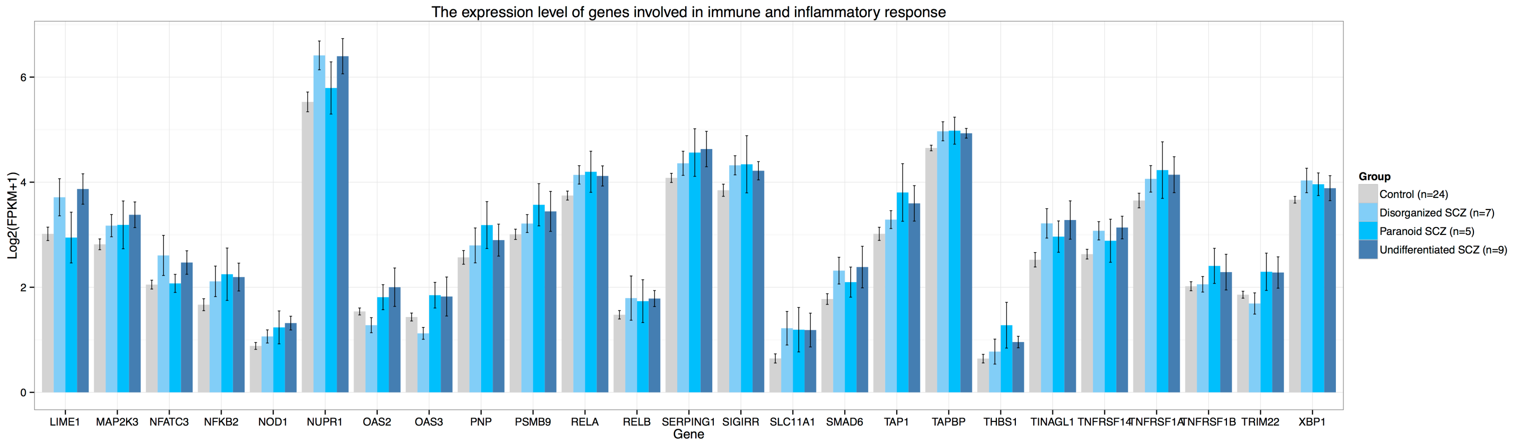

Figure S5. Barplot showing expression levels of up-regulated genes in blood vessel development.

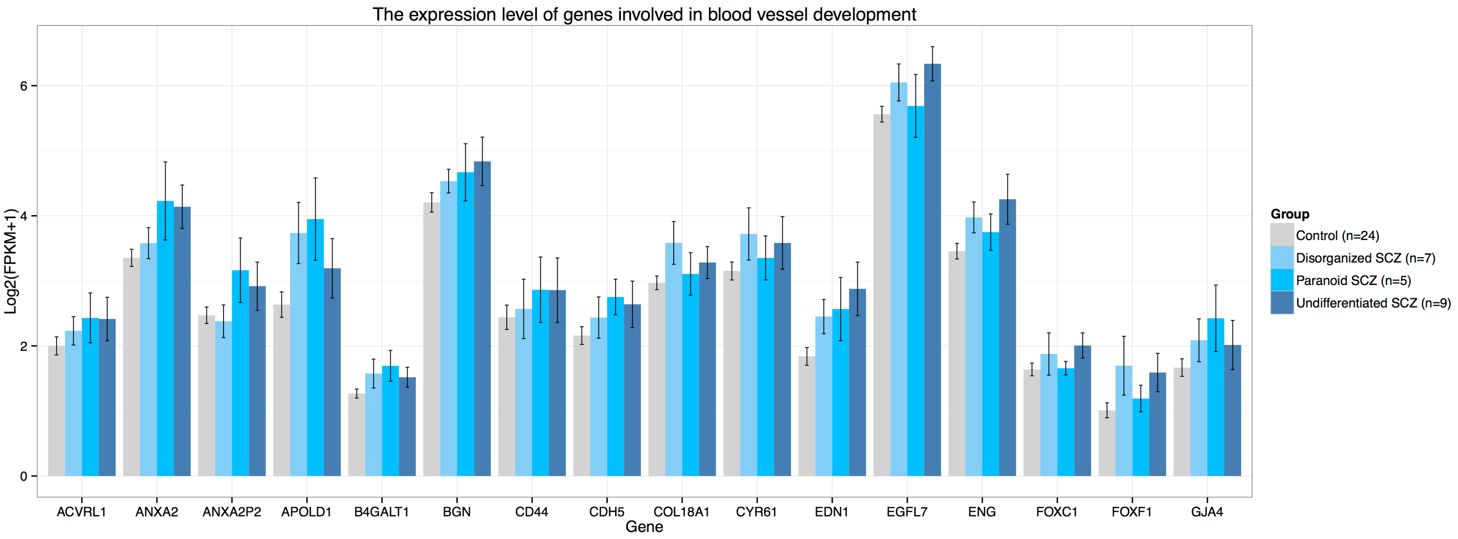

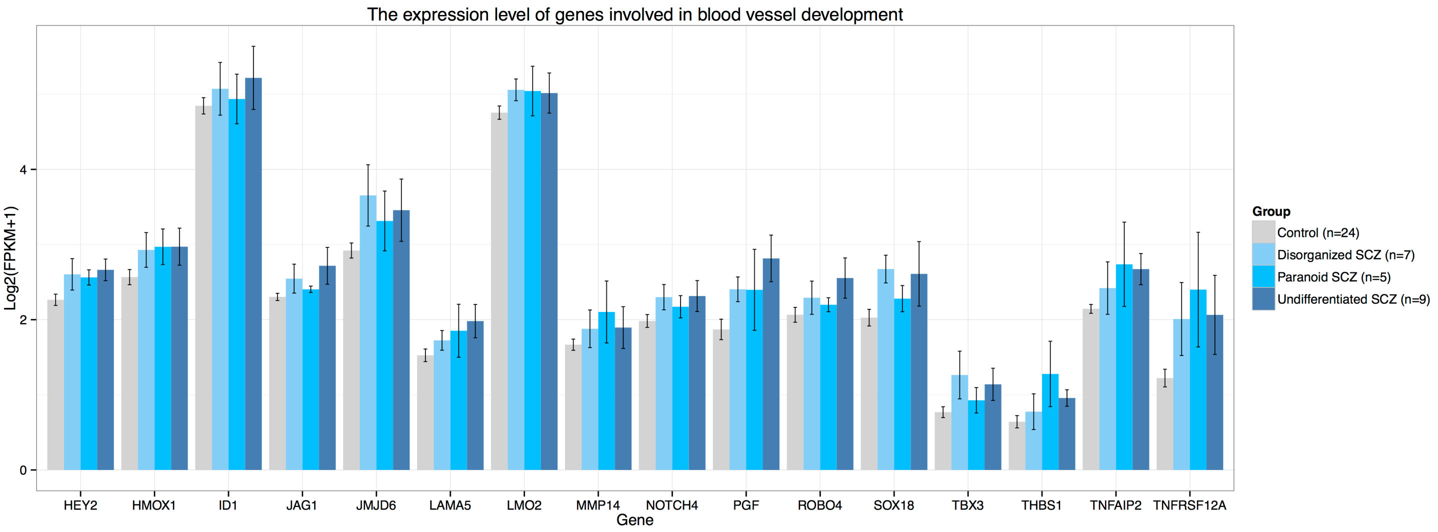

Figure S6. Barplot showing expression levels of down-regulated genes in synaptic transmission.

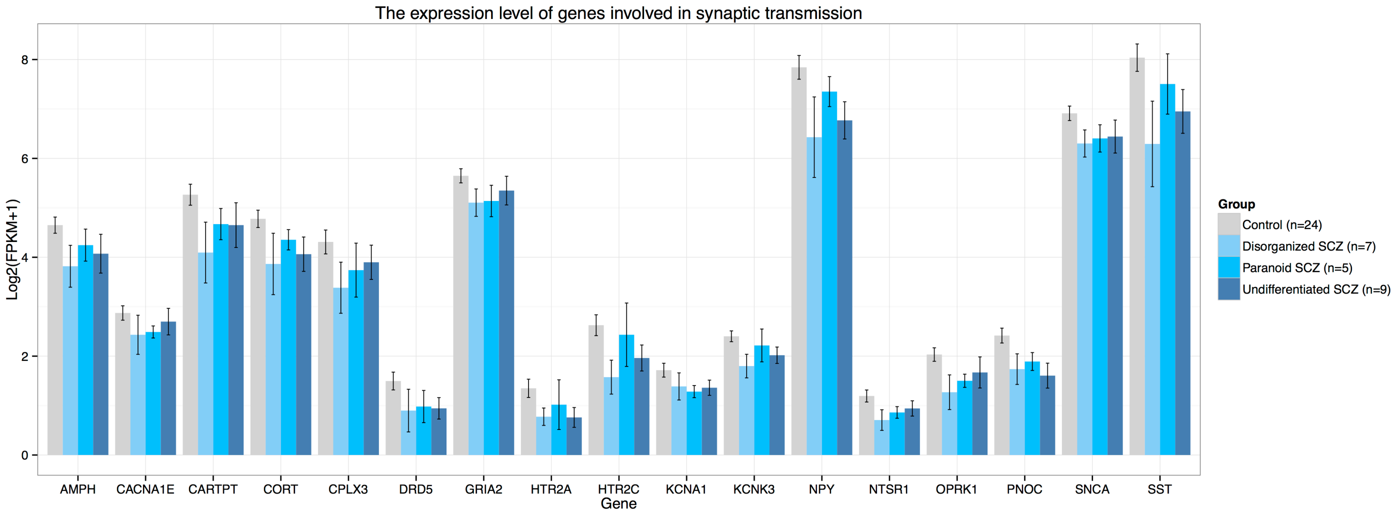

Figure S7. Bar plot showing expression levels of genes differentially expressed among subtypes of schizophrenia.

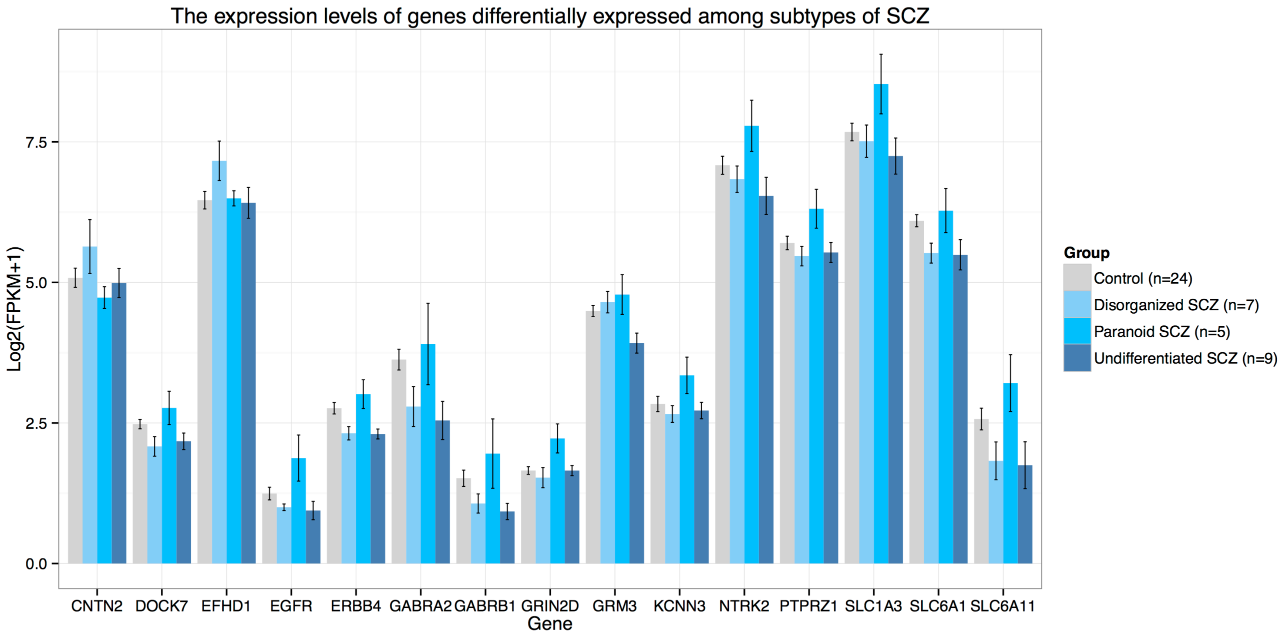

1. Guidotti A, Auta J, Davis JM, Di-Giorgi-Gerevini V, Dwivedi Y, Grayson DR, et al. (2000): Decrease in reelin and glutamic acid decarboxylase67 (GAD67) expression in schizophrenia and bipolar disorder: a postmortem brain study. *Archives of general psychiatry*. 57:1061-1069.
2. Weidenhofer J, Bowden NA, Scott RJ, Tooney PA (2006): Altered gene expression in the amygdala in schizophrenia: up-regulation of genes located in the cytomatrix active zone. *Molecular and cellular neurosciences*. 31:243-250.
3. Arion D, Unger T, Lewis DA, Levitt P, Mirnics K (2007): Molecular evidence for increased expression of genes related to immune and chaperone function in the prefrontal cortex in schizophrenia. *Biological psychiatry*. 62:711-721.
4. Saetre P, Emilsson L, Axelsson E, Kreuger J, Lindholm E, Jazin E (2007): Inflammation-related genes up-regulated in schizophrenia brains. *BMC psychiatry*. 7:46.
5. Choi KH, Elashoff M, Higgs BW, Song J, Kim S, Sabunciyan S, et al. (2008): Putative psychosis genes in the prefrontal cortex: combined analysis of gene expression microarrays. *BMC psychiatry*. 8:87.
6. Hashimoto T, Arion D, Unger T, Maldonado-Aviles JG, Morris HM, Volk DW, et al. (2008): Alterations in GABA-related transcriptome in the dorsolateral prefrontal cortex of subjects with schizophrenia. *Molecular psychiatry*. 13:147-161.
7. Wu JQ, Wang X, Beveridge NJ, Tooney PA, Scott RJ, Carr VJ, et al. (2012): Transcriptome sequencing revealed significant alteration of cortical promoter usage and splicing in schizophrenia. *PloS one*. 7:e36351.
8. Xu J, Sun J, Chen J, Wang L, Li A, Helm M, et al. (2012): RNA-Seq analysis implicates dysregulation of the immune system in schizophrenia. *BMC genomics*. 13 Suppl 8:S2.
9. Fillman SG, Cloonan N, Catts VS, Miller LC, Wong J, McCrossin T, et al. (2013): Increased inflammatory markers identified in the dorsolateral prefrontal cortex of individuals with schizophrenia. *Molecular psychiatry*. 18:206-214.

10. Hwang Y, Kim J, Shin JY, Kim JI, Seo JS, Webster MJ, et al. (2013): Gene expression profiling by mRNA sequencing reveals increased expression of immune/inflammation-related genes in the hippocampus of individuals with schizophrenia. *Translational psychiatry*. 3:e321.
11. Mirnics K, Middleton FA, Lewis DA, Levitt P (2001): Analysis of complex brain disorders with gene expression microarrays: schizophrenia as a disease of the synapse. *Trends in neurosciences*. 24:479-486.
12. Fatemi SH, Stary JM, Earle JA, Araghi-Niknam M, Eagan E (2005): GABAergic dysfunction in schizophrenia and mood disorders as reflected by decreased levels of glutamic acid decarboxylase 65 and 67 kDa and Reelin proteins in cerebellum. *Schizophrenia research*. 72:109-122.
13. Guillozet-Bongaarts AL, Hyde TM, Dalley RA, Hawrylycz MJ, Henry A, Hof PR, et al. (2014): Altered gene expression in the dorsolateral prefrontal cortex of individuals with schizophrenia. *Molecular psychiatry*. 19:478-485.
